# Kava Herb‐Induced Liver Injury as Verified by the Updated RUCAM

**DOI:** 10.1155/crgm/3914876

**Published:** 2025-12-29

**Authors:** Sahan Withanage, Carl Cosgrave, Shoa Zafir, Eliza Flanagan, Phillip Te, Samuel Hall

**Affiliations:** ^1^ University Hospital Geelong, Barwon Health, Geelong, Victoria, Australia, barwonhealth.org.au; ^2^ Department of Medicine, Deakin University, Geelong, Victoria, Australia, deakin.edu.au; ^3^ Australian Clinical Labs, Geelong, 3220, Australia

**Keywords:** drug-induced liver injury, hepatotoxicity, kava, liver

## Abstract

A temporal relationship between liver enzyme derangement and an herbal remedy warrants further assessment for herb‐induced liver injury (HILI). Here, we describe the use of kava, a drink traditionally consumed in Pacific Island cultures, causing acute ALT and AST elevation as assessed by an updated RUCAM score of 7. The increasing use of kava in Western society should prompt clinicians to be more aware of this rare cause of HILI. A 46‐year‐old man was referred to the emergency department with a 3‐week history of fatigue, right upper quadrant pain, and profound transaminitis. He commenced kava 10 g daily 5 weeks prior to aid sleep, which was ceased 2 weeks prior due to his biochemical derangement. Blood tests revealed an ALT of 1546 U/L and an AST of 920 U/L. An autoimmune screen, viral serology, and liver ultrasound showed no abnormalities. A liver biopsy revealed foci of hepatocellular necrosis with scattered ballooning degeneration and apoptotic bodies in the parenchyma, but normal underlying hepatic parenchyma without steatosis. Following cessation of kava, the liver enzymes improved without any other intervention. He was monitored as an outpatient and had no recurrence. The incidence of kava HILI may increase with its marketing; its exact mechanism is unknown. Ultimately, further research is needed to identify the pathogenesis of kava HILI. HILI is a significant cause of transaminitis, and clinicians should remain vigilant in patients presenting with nonspecific symptoms and a negative liver screen.

## 1. Introduction

Herb‐induced liver injury (HILI) is a relatively common yet challenging diagnosis, often with the temporal relationship to the precipitating drug being the only initial clue. Kava is a South Pacific psychotropic plant medicine with anxiolytic activity through GABA modulation, monoamine oxidase B inhibition, and noradrenaline and dopamine reuptake inhibition [1]. It is derived from the roots of the plant *Piper methysticum*. Polynesian, Melanesian, and Micronesian people have used it to prepare a traditional beverage often used in solemn rituals [2]. Recently, the marketing of kava as an herbal remedy for anxiety syndromes has led to increased consumption in Western societies. However, due to inconsistent regulation, variable preparations and dosages of kava are available. Current evidence supports the efficacy of kava in the treatment of anxiety, though regular liver function tests (LFTs) are advised [1]. While it is well established that kava can cause hepatotoxicity, the specific ingredient or mechanism responsible remains unclear. Despite its recognition, there are relatively few case reports in the literature documenting kava‐induced liver injury. Here, we present a case of acute transaminitis following 5 weeks of kava use for insomnia.

## 2. Case

A 46‐year‐old Australian‐born man was referred to the emergency department by his local medical officer (LMO) with a 3‐week history of fatigue, dull right upper quadrant (RUQ) pain, and profound, progressive LFT derangement. His past medical history included hypertension, depression, gastroesophageal reflux, active smoking with a 30 pack‐year history, and a previous diagnosis of alcohol misuse disorder (now abstinent for 12 months). His prescribed medications included perindopril, sertraline, naltrexone, and pantoprazole, and he had recently commenced paracetamol 1 g twice daily for his RUQ pain, which was ceased on admission. He had commenced kava (10 g daily) five weeks prior to his presentation to assist with sleep, which was stopped two weeks prior by his LMO after discovering his LFT derangement. He was not taking any other medications or supplements. He was in a long‐term heterosexual relationship and had no history of at‐risk sexual behaviors or intravenous drug use. He had no personal or family history of liver disease.

On examination, there was no evidence of jaundice or peripheral stigmata of chronic liver disease. His abdomen was soft and nontender, and there was no evidence of hepatosplenomegaly. His full blood count was unremarkable. His biochemistry revealed an ALT of 1546 U/L (5–40 U/L) and an AST of 920 U/L (5–35 U/L). His GGT was 493 U/L (5–50 U/L), ALP 194 U/L (30–110 U/L), and bilirubin 11 μmol/L (3–20 μmol/L). Other markers of synthetic liver function (Table 1) along with renal function were normal. Autoimmune screen including antismooth muscle antibody, antimitochondrial antibody, antinuclear antibody, liver–kidney microsomal Type 1 antibody, and immunoglobulins was unremarkable. Viral hepatitis (B and C) and human immunodeficiency virus serology were also negative. Epstein–Barr and cytomegalovirus testing revealed evidence of previous exposure without active infection. Paracetamol level was < 20 μmol/L (60–120 μmol/L). A liver ultrasound demonstrated normal liver architecture with a normal biliary tree and no evidence of portal or hepatic vein thrombosis.

**Table 1:** Liver function tests and markers of synthetic function.

	Presentation	D3 admission	6 weeks postdischarge	3 months postdischarge
ALP (30–110 U/L)	194	161	156	225
GGT (5–50 U/L)	493	473	298	447
AST (3–35 U/L)	920	608	786	249
ALT (5–40 U/L)	1546	1249	543	159
Bilirubin (3–20 μmol/L)	11	12	10	9
Albumin (35–50 g/L)	39	31	39	36
INR	1.1	1.0	1.1	1.1
Iron studies				
Ferritin (30–400 μg/L)	3154			
Iron (μmol/L)	23.2			
Saturation (15%–50%)	42			

**Figure 2:**
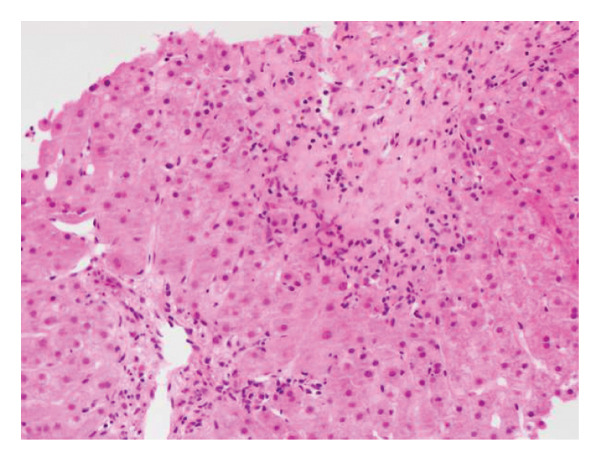
H&E‐stained section demonstrates interface hepatitis with mixed inflammatory cell infiltrate, including plasma cells, eosinophils, and neutrophils.

After other differentials for acute profound transaminitis (ALT > 1000) were excluded, a working diagnosis of acute kava HILI was made considering the temporal relationship between the onset of kava ingestion and development of symptoms. Paracetamol was considered an unlikely culprit due to a serum level of < 20 μmol/L and its commencement post the onset of RUQ pain. The patient underwent a liver biopsy which revealed foci of hepatocellular necrosis with scattered ballooning degeneration and apoptotic bodies in the parenchyma but normal underlying hepatic parenchyma without evidence of steatosis to imply chronicity (Figures 1 and 2). There was also mixed periportal inflammation but a normal bile duct caliber with no evidence of cholestasis. Paracetamol remains a confounding factor as there is little inflammation relative to zones of necrosis. These findings led to a diagnosis of HILI precipitated by kava. Following cessation of the herb, the patient’s liver enzymes improved without any intervention. He was monitored as an outpatient and has not had any further episodes of LFT derangement. The updated RUCAM yielded a score of 7, indicating probable causality [3]. Points were obtained for a hepatocellular injury pattern that was confirmed with onset between 5 and 90 days of ingestion, known hepatotoxicity of kava, a supportive biopsy, and the ruling out of nondrug causes [3].

Figure 1(a) Hematoxylin and eosin (H&E)–stained section demonstrates hepatocyte necrosis and secondary ductal proliferation (white arrow) associated with inflammation, compared to adjacent unaffected parenchyma (black arrow) and (b) corresponding reticulin‐stained section highlights hepatocellular necrosis (white arrow) compared to adjacent viable liver parenchyma (black arrow).

**Figure:**
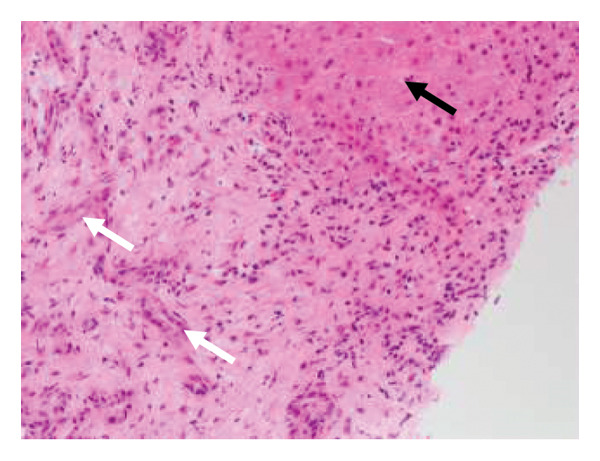
(a)

**Figure:**
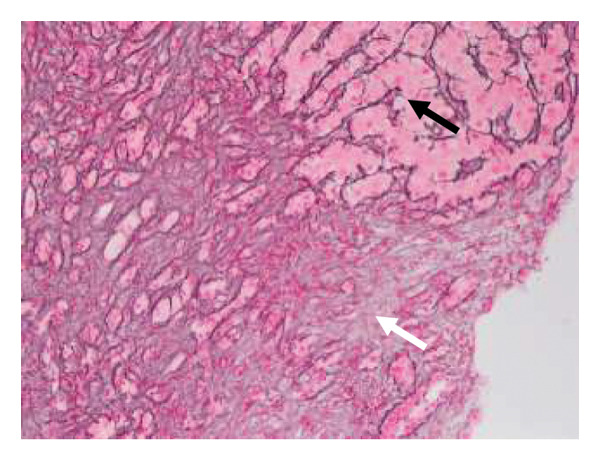
(b)

## 3. Discussion

Kava kava (more commonly known as kava) is an herbal supplement derived from the roots of the *P. methysticum* plant, indigenous to the southern and western Pacific. It has been used for centuries in Pacific Island cultures as a recreational and ceremonial drink, as the kavalactones (the principal pharmacologically active components of kava) provide effects of relaxation, sociability, and euphoria. More recently, it has been marketed in more concentrated forms of herbal medications in Western society to treat anxiety and insomnia [4]. Although rare in conventional dosing, kava does seem to have abuse potential. Clinically apparent liver injury due to kava supplementation is rare. Still, it is increasingly recognized as a side effect, leading to restrictions in many countries, including Germany, the United Kingdom, Switzerland, France, Canada, and Japan. In Australia, kava was banned in New South Wales from 2007 to 2021 and prohibited in the Northern Territory since 2020 [5].

Patients with kava‐related HILI typically present with primary complaints of fatigue and nausea, with laboratory testing revealing profound transaminitis with jaundice developing after weeks to months of sustained use [6]. Histology usually reveals focal hepatocellular necrosis, lobular inflammation, and intrahepatic cholestasis [7, 8]. While most cases of liver injury tend to be self‐limiting once the agent has been removed, rare cases have been reported of fulminant hepatic failure requiring liver transplantation or death [6, 9]. In milder cases, the liver injury typically subsides within 1 to 3 months of discontinuation of the product. Rechallenge has been reported to lead to prompt recurrence and should be avoided [7, 10].

The exact mechanism of hepatotoxicity is not well understood but is thought to be idiosyncratic or immunologically mediated [6]. Overdose has also been suggested, with substantial variations in dosing recommendations and no well‐established toxic dose guidelines. The pharmacological properties of kava are attributed to a poorly characterized group of compounds termed kavalactones. While these compounds are not overtly cytotoxic [11], kavalactones have been shown to inhibit cytochrome P450, which is responsible for a significant portion of pharmaceutical metabolism in modern medicine, raising the possibility that drug–drug interactions play a key role in pathogenesis [12]. Given the lack of reports in Polynesian populations, genetic susceptibility has also been raised but not substantiated [7]. Newer extraction techniques used in herbal formulations (using ethanol or acetone for higher yield) have also been implicated [13, 14]. The fact that kava‐induced hepatotoxicity was not reported until 1998 may further support the argument that inferior (extracted) quality or production methods in the Western herbal medicine market may contribute [15]. Other risk factors identified have been overdose, prolonged treatment, and comedication with other synthetic drugs or supplements [7].

Ultimately, further research is needed to identify the pathogenesis of kava‐induced liver injury. Herbal supplementation remains a significant cause of clinically apparent HILI, and clinicians should remain vigilant in patients presenting with nonspecific symptoms and a negative liver screen.

## Disclosure

This study was performed as part of the author’s employment at Barwon Health.

## Conflicts of Interest

The authors declare no conflicts of interest.

## Author Contributions

Dr Sahan Withanage: literature review, preparation of the manuscript, and review of the manuscript (lead).

Dr Carl Cosgrave: preparation of the manuscript, literature review, and patient consent.

Dr Shoa Zafir: supply of histological images.

Dr Phillip Te: review of the manuscript.

Dr Eliza Flanagan: review of the manuscript.

Dr Samuel Hall: review of the manuscript prior to submission and direct involvement in patient care.

## Funding

This research received no funding.

## Data Availability

Data sharing is not applicable to this article as no datasets were generated or analyzed during the current study.
